# Inflammation Links Cardiac Injury and Renal Dysfunction: A Cardiovascular Magnetic Resonance Study

**DOI:** 10.31083/j.rcm2504148

**Published:** 2024-04-22

**Authors:** Xiaohui Xie, Jiahong Chen, Lei Yu, Jianzhong Sun, Chengchen Zhao, Qingqing Duan

**Affiliations:** ^1^Department of Nephrology, Zhejiang Hospital, 310009 Hangzhou, Zhejiang, China; ^2^Department of Nephrology, Xiamen Hongai Hospital, 361000 Xiamen, Fujian, China; ^3^Department of Radiology, The Second Affiliated Hospital, Zhejiang University School of Medicine, 310009 Hangzhou, Zhejiang, China; ^4^Department of Cardiovascular disease, The Second Affiliated Hospital, Zhejiang University School of Medicine, 310009 Hangzhou, Zhejiang, China

**Keywords:** heart failure, T1 mapping, cardiorenal syndrome, inflammation, cardiovascular magnetic resonance

## Abstract

**Background:**

Inflammation is essential in cardiorenal syndrome, however there is still a lack of evidence proving the interaction between cardiac injury, renal dysfunction and the inflammatory response. This study aimed to illustrate the association between renal dysfunction and cardiac injury with a specific focus on the role of inflammation.

**Methods:**

A single-center, retrospective study included patients with heart failure admitted to the cardiovascular department from September 2019 to April 2022. Patients received cardiovascular magnetic resonance (CMR) imaging (T1 mapping and late gadolinium enhancement (LGE)). Demographic, creatinine and native T1 were analyzed using pearson correlation, linear regression and adjusted for confounders. Interaction and subgroup analysis were performed.

**Results:**

Finally, 50 validated heart failure (HF) patients (age 58.5 ± 14.8 years; 78.0% men) were included. Cardiac global native T1 for the high estimated glomeruar filtration rate (eGFR) group was 1117.0 ± 56.6 ms, and for the low eGFR group was 1096.5 ± 61.8 ms. Univariate analysis identified global native T1 (β = 0.16, 95% confidence interval (CI): 0.04–0.28, *p* = 0.014) and C-reactive protein (CRP) (β = 0.30, 95% CI: 0.15–0.45, *p* < 0.001) as determinants of creatinine. Multivariable linear regression analysis identified global native T1 (β = 0.12, 95% CI: 0.01–0.123, *p* = 0.040) as a determinant of creatinine while age and diabetes were adjusted. Significant interactions between CRP and global native T1 in relation to creatinine level (*p* for interaction = 0.005) were identified.

**Conclusions:**

Kidney dysfunction was associated with cardiac injury and inflammation, respectively. The interaction between myocardial injury and kidney dysfunction is contingent on the severity of the inflammatory response. Further studies were needed to identify the mechanisms of the inflammatory response in cardiorenal syndrome.

## 1. Introduction

Heart failure often coexists with several comorbidities of which chronic kidney disease (CKD) is a strong predictor of poor outcomes [1, 2, 3]. The interaction between heart and kidney dysfunction is both complex and bi-directional, and has been referred to as cardiorenal syndrome. Three mechanisms have been proposed to contribute to the development of cardiorenal syndrome, including hemodynamic, hormonal, and cardiovascular disease-related factors [4, 5]. Systemic and chronic low-grade inflammation increased expression of interleukin (IL)-1β, tumor necrosis factor (TNF)-α, and other cytokines leads to changes in nitric oxide production, as well as alterations in cardiac and kidney; it is considered to be a key driver of both CKD and cardiac injury, and may serve as a surrogate therapeutic target [4, 6, 7].

However, detecting subtle pathological changes during the progress of cardiorenal syndrome can be challenging due to the limited accuracy and specificity of current biomarkers [1, 8, 9]. Cardiovascular imaging may provide valuable insights into organ damage and inflammation in this context. T1 mapping, assessed by cardiovascular magnetic resonance (CMR) imaging, is a surrogate biomarker of myocardial fibrosis burden. Previous studies have demonstrated the association between T1 mapping and worsening kidney function, suggesting that it might be a practical tool in assessing the presence and progression of cardiorenal syndrome [10, 11, 12, 13, 14]. As indicators of renal dysfunction, creatinine and eGFR were frequently used.

This study aimed to illustrate the association between renal dysfunction and cardiac injury with a specific focus on the role of inflammation, as represented by C-reactive protein (CRP). By comprehensively examining the association between cardiac injury and renal function in heart failure patients, we hope to gain a better understanding of inflammatory damage in the cardiorenal syndrome.

## 2. Methods

### 2.1 Study Design and Clinical Setting

**Fig. 1.**
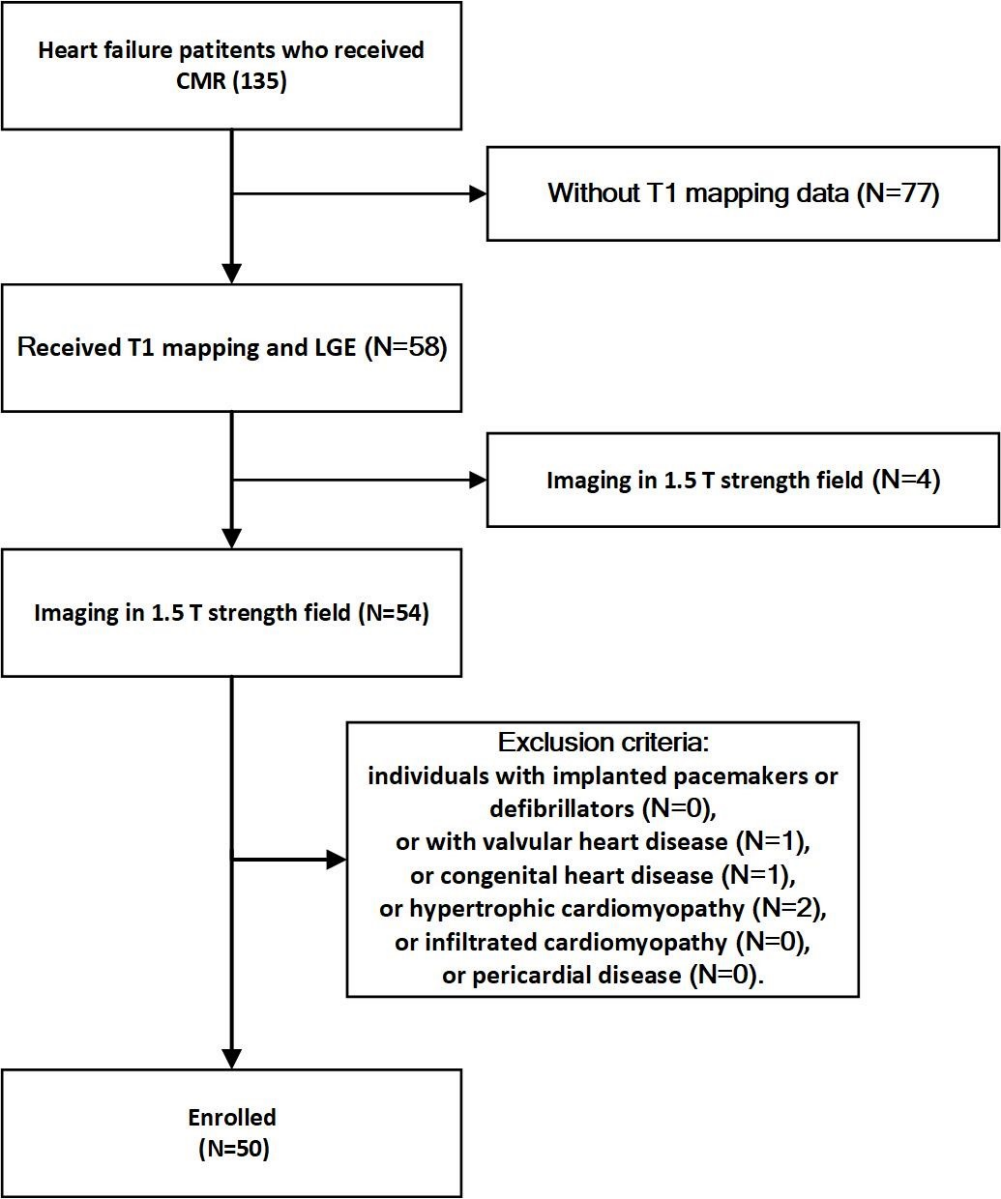
**Flow chart of patients selection**. CMR, magnetic resonance image; LGE, late gadolinium enhancement.

It was a retrospective, single-center study approved by the institutional review board. Informed consent was obtained from patients for this study (Num-2020-1052). Participants received CMR at our institution between September 2019 to April 2022. The inclusion criteria were as follows: heart failure with symptomatic clinical syndrome with or without elevated N-terminal pro-B type natriuretic peptide (NT-proBNP); received cardiovascular magnetic resonance imaging (T1 mapping and LGE (late gadolinium enhancement)). Exclusion criteria for this study were defined as follows: individuals with implanted pacemakers or defibrillators, hypertrophic cardiomyopathy, infiltrated cardiomyopathy, valvular heart disease, congenital cardiac disease, or pericardial disease. Details were summarized in Fig. 1. This study complied with the Strengthening the Reporting of Observational Studies in Epidemiology (STROBE) reporting guideline.

Demographics and laboratory data were recorded from the electronic medical system. Laboratory items were listed below: CRP, Hematocrit (HCT), glycated hemoglobin (HbA1c), low-density lipoprotein (LDL), D-dimer, NT-proBNP, white blood cell count (WBC), alanine aminotransferase (ALT), cardiac troponin I (cTnI), serum creatinine (Cr), lymphocyte count and ratio, neutrophil count and ratio. The patients were divided according to eGFR ≥75 mL/min/1.73 $m^{2}$.

### 2.2 CMR Image Acquisition and Analysis 

All magnetic resonance imaging (MRI) data were acquired on a 1.5 T MRI system (Aera, Siemens Healthineers). Cine images with retrospective electrocardiogram (ECG) gating during a breath-hold were adopted from a balanced steady-state free precession sequence. The imaging parameters were as follows: the average temporal resolution 45.6 ms. 9–12 slices of short-axis views (8 mm thickness) and three long-axis views were obtained using the following sequence parameters: flip angle 35°, echo time (TE) 1.12 ms, repetition time (TR) 2.60 ms, and average in-plane resolution 2.10 × 1.40 ${\mathrm{mm}}^{2}$.

LGE images were acquired 10 minutes after administration of gadolinium agent using a gradient-spoiled, turbo-fast, low-angle shot sequence with a phase-sensitive inversion recovery sequence. The images were obtained in the long-axis views (2-chamber and 4-chamber), as well as a series of contiguous 6-mm LV (left ventricle) short-axis slices that covered the entire LV. The imaging parameters were as follows: TR/TE, 700 ms/1.28 ms; time of inversion (TI) 350 ms; flip angle 40°, spatial resolution 1.8 × 1.8 × 8 ${\mathrm{mm}}^{3}$.

The pre-contrast modified look-locker inversion recovery (MOLLI) images followed the 5(3)3 protocol during a breath-hold. Post-contrast MOLLI images followed the 4(1)3(1)2 protocol during a breath-hold 10 min after contrast administration. T1 images were acquired from 3 short-axis slices (basal, mid, and apical). The apical slice was chosen as the most proximal slice of the apical segment to avoid partial volume averaging. Imaging parameters were: TR = 2.60 ms, TE = 1.12 ms, flip angle (FA) = 35°, in-plane resolution = 2.10 × 1.41 ${\mathrm{mm}}^{2}$, slice thickness = 8 mm.

An experienced physician used CVI42 version 5.13.4 (Circle Cardiovascular Imaging Inc., Calgary, Canada) to analyze MRI images. The measures included LV end-diastolic volume, LV end-systolic volume, stroke volume, LV mass, and LV ejection fraction (EF), right ventricle (RV) EF, left atrium volume. Global longitudinal strain (GLS, %), global radial strain (GRS, %), and global circumferential strain (GCS, %) were also calculated through CVI42.

T1 relaxation times were measured using regions of interest drawn in the short-axis views. Regions of interest avoided the papillary muscles and border of blood partial volume effect. Averaged T1 values of the short-axis slices were calculated, and global T1 values were defined as the mean value.

An extracellular volume (ECV) map was generated from a native T1 map and a post-contrast T1 map through CVI42. It was calculated using the mean segmental pixel value from the MOLLI ECV maps and using the formula below:

$$\begin{array}{c} \mathrm{ECV}=\left(\Delta \,{\mathrm{R1}}_{\text{myocardium }}/\Delta \,{\mathrm{R1}}_{\text{blood }}\right)*\left(1-\text{ hematocrit }\right)\\ \mathrm{R1}=1/\mathrm{T1}\,\text{ time } \end{array}$$

Intra-observer variabilities for T1 values of the LV segments were assessed in a randomly selected 10 subjects.

### 2.3 Statistical Analyses

Categorical and consecutive data were presented as number (%), mean ± standard deviation (data fitted normal distribution), or media, quartile (data did not fit normal distribution). An unpaired *t*-test or Kruskal-Wallis test was adopted to evaluate differences between means as appropriate. Pearson correlation was adopted for correlation analysis between variables. Univariate and Multivariable linear regression was carried out to investigate the association of creatinine with native T1 and CRP. Interaction analysis was conducted. Intra-observer repeatability was assessed for T1 mapping using the intraclass correlation coefficient. Statistical significance was defined as *p* < 0.05. Statistical analysis was performed using the R package (version 4.11, R foundation for statistical computing, Vienna, Austria).

## 3. Results

### 3.1 Demographics and Clinical Status

Baseline demographics of all heart failure patients are summarized in Table 1. Non-significant differences were observed between the two groups regarding age, sex, blood pressure, and heart rate except for body mass index (BMI). Compared to patients with eGFR ≥75 mL/min/1.73 $m^{2}$ (high eGFR group), patients with eGFR <75 mL/min/1.73 $m^{2}$ (low eGFR group) had higher lymph count, LA volume, LV mass, and body mass index (*p* < 0.05). The two groups were similar in New York Heart Association class, heart failure biomarker, and medication history. A significant difference in comorbidity including coronary artery disease, atrial fibrillation, and hypertension was not identified between the two groups (Table 1).

**Table 1.** **Baseline characteristics of heart failure patients**.

		High eGFR group	Low eGFR group	*p*
		(N = 29)	(N = 21)	
Demographics				
	Sex (male)	20 (69.0%)	19 (90.5%)	0.092
	Age	58.7 (14.8)	58.2 (15.2)	0.901
	Weight (kg)	59.2 (8.3)	83.6 (10.9)	<0.001
	Height (cm)	163.4 (7.7)	168.4 (7.2)	0.023
	Body mass index (${\mathrm{kg/m}}^{2}$)	22.2 (2.6)	29.1 (3.1)	<0.001
	Systolic BP (mmHg)	113.7 (17.8)	110.3 (11.5)	0.417
	Diastolic BP (mmHg)	67.8 (12.4)	67.7 (16.4)	0.985
	Heart rate	77.0 [62.0; 85.0]	77.0 [73.0; 102.0]	0.226
	Smoke	3 (10.3%)	7 (33.3%)	0.073
	Alcohol	4 (13.8%)	5 (23.8%)	0.464
	Hypertension	13 (44.8%)	12 (57.1%)	0.567
	Diabetes	7 (24.1%)	3 (14.3%)	0.488
	Coronary artery disease	11 (37.9%)	11 (52.4%)	0.467
	Atrial fibrillation	4 (13.8%)	5 (23.8%)	0.464
	Thyroid disease	1 (3.4%)	0 (0.0%)	0.999
	Stroke	2 (6.9%)	1 (4.8%)	0.999
	NYHA >2	7 (24.1%)	4 (19.0%)	0.836
	ARNi	20 (69.0%)	19 (90.5%)	0.092
	Beta blocker	22 (75.9%)	19 (90.5%)	0.271
	MRA	23 (79.3%)	17 (81.0%)	0.999
	Diuretics	20 (69.0%)	17 (81.0%)	0.531
	Digoxin	2 (6.9%)	0 (0.0%)	0.503
	Amiodarone	2 (6.9%)	3 (14.3%)	0.638
	CCB	0 (0.0%)	1 (4.8%)	0.420
	Anti platelet	15 (51.7%)	10 (47.6%)	0.999
	Anti coagulation	7 (24.1%)	4 (19.0%)	0.741
	Statin	19 (65.5%)	12 (57.1%)	0.759
Laboratory tests				
	Hct (L/L)	41.6 (5.7)	42.8 (5.1)	0.413
	HbA1c (%)	5.9 [5.5; 6.4]	6.0 [5.5; 6.7]	0.595
	D-dimer (ug/L)	330.0 [220.0; 1020.0]	400.0 [230.0; 590.0]	0.774
	Alanine aminotransferase (mmol/L)	29.0 [26.0; 36.0]	31.0 [27.0; 36.0]	0.984
	NT-proBNP (pg/mL)	1184.0 [216.0; 2685.0]	1020.0 [402.0; 1856.0]	0.992
	cTnI (ng/mL)	<0.1 [<0.1; <0.1]	<0.1 [<0.1; <0.1]	0.738
	Creatinine (umol/L)	81.0 [63.0; 97.0]	81.0 [75.0; 90.0]	0.776
	eGFR (mL/min/1.73 $m^{2}$)	80.8 (8.3)	56.4 (10.9)	<0.001
	CRP (mg/L)	5.0 [3.4; 21.7]	5.0 [3.4; 9.3]	0.633
	WBC (${\mathrm{10}}^{9}$/L)	6.2 (1.8)	6.6 (2.0)	0.455
	Lymphocyte count (${\mathrm{10}}^{9}$/L)	1.3 [1.0; 1.7]	1.5 [1.4; 2.3]	0.014
	Lymphocyte ratio (%)	25.2 (8.7)	27.2 (10.8)	0.475
	Neutrophil count (${\mathrm{10}}^{9}$/L)	3.8 [2.8; 4.9]	4.5 [3.7; 5.3]	0.194
	Neutrophil ratio (%)	64.8 [59.2; 69.4]	68.7 [56.2; 72.8]	0.768
CMR parameters				
	LV end-diastolic volume (mL)	242.1 (83.2)	274.6 (73.6)	0.152
	LV end-systolic volume (mL)	178.8 (82.2)	215.0 (78.7)	0.123
	LV EF (%)	27.1 [17.4; 36.8]	22.4 [13.1; 33.7]	0.382
	Stroke volume (mL)	58.1 [41.2; 74.8]	59.0 [42.4; 79.2]	0.875
	Cardiac mass (g)	118.9 [92.8; 145.8]	144.4 [124.5; 166.2]	0.006
	RV EF (%)	35.5 (15.6)	28.5 (14.8)	0.112
	LA volume (mL)	82.7 [74.8; 98.1]	121.2 [83.7; 152.4]	0.006
	LV GCS (%)	–8.4 [–10.2; –6.5]	–5.8 [–9.5; –4.5]	0.135
	LV GRS (%)	10.7 [8.2; 14.2]	8.1 [5.3; 14.3]	0.205
	LV GLS (%)	–8.1 [–10.6; –6.2]	–6.7 [–10.3; –5.4]	0.326
	LGE (positive)	22 (75.9%)	14 (66.7%)	0.692
	Native T1 (ms)	1117.0 (56.6)	1096.5 (61.8)	0.236
	Post T1 (ms)	274.6 (71.2)	310.6 (49.8)	0.041
	Extracellular volume (%)	39.1 (9.5)	35.4 (10.2)	0.203

All values are presented as the means (SD) or n (%) or as the median [interquartile range]. N, number of individuals; CRP, c-reactive protein; HbA1c, glycated hemoglobin; eGFR, estimated glomeruar filtration rate; BP, blood pressure; NT-proBNP, N-terminal pro-B type natriuretic peptide; WBC, white blood cell count; NYHA, New York Heart Association; ARNi, angiotensin receptor neprilysin inhibitor; cTnI, cardiac troponin I; MRA, mineralcorticoid recept antagonist; CCB, calcium channel blocker; Hct, hematocrit value; CMR, magnetic resonance image; LV, left ventricle; EF, ejection fraction; RV, right ventricle; LA, left atrium; LGE, late gadolinium enhancement; GLS, global longitudinal strain; GRS, global radial strain; GCS, global circumferential strain.

There was no significant difference in LV end-diastolic volume, LV EF, RV EF, and myocardial strain (Table 1). Over 60% of all patients had myocardial scar with no overall difference between the two groups for the LGE existence (*p* = 0.692). Significant differences were observed between the two groups, and both the high eGFR group (eGFR ≥75 mL/min/1.73 $m^{2}$) and the low eGFR group (eGFR <75 mL/min/1.73 $m^{2}$) patients’ groups regarding myocardial post T1 which were higher in the high eGFR group (high eGFR: 274.6 ± 71.2 ms vs low eGFR: 310.6 ± 49.8 ms, *p* = 0.041). Non-significant differences of native T1 (high eGFR: 1117.0 ± 56.6 ms vs low eGFR: 1096.5 ± 61.8 ms, *p* = 0.236) and ECV (high eGFR: 39.1 ± 9.5 ms vs low eGFR: 35.4 ± 10.2 ms, *p* = 0.203) were observed between the two groups.

### 3.2 Correlation between Inflammation, Cardiac Damage, and Renal Dysfunction 

**Fig. 2.**
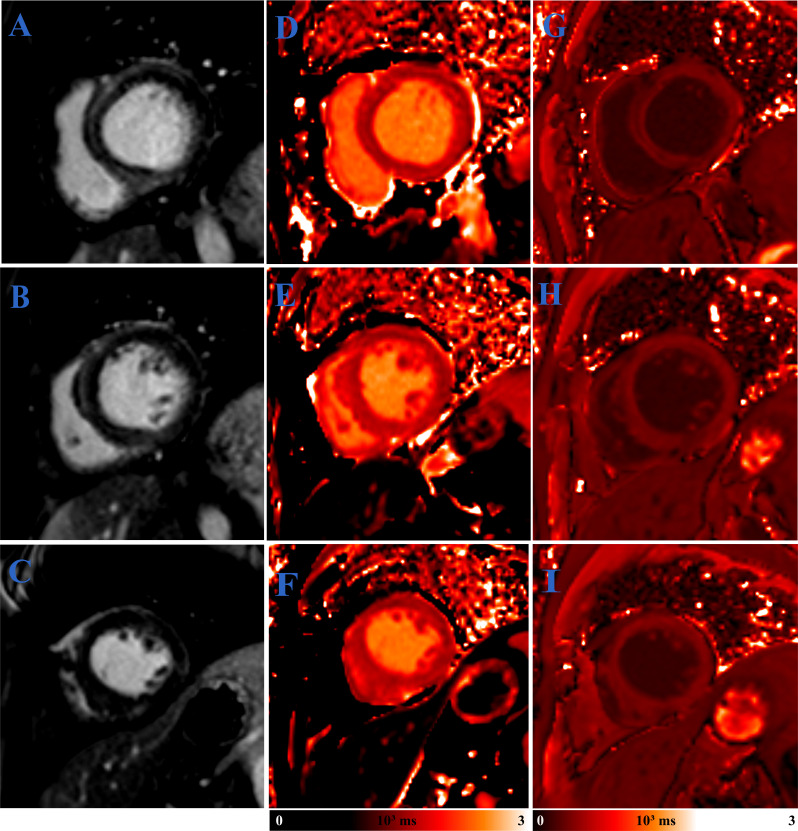
**Typical cardiovascular magnetic resonance images from a 58-year-old male patient with chronic kidney disease**. PSIR, LGE images (A–C), native T1 (D–F) and post T1 images (G–I) were displayed separately in different columns. Segments from basal to apical were displayed in rows. Color bars were added separately for images from (D–F) and (G–I). PSIR, phase-sensitive inversion recovery; LGE, late gadolinium enhancement.

**Fig. 3.**
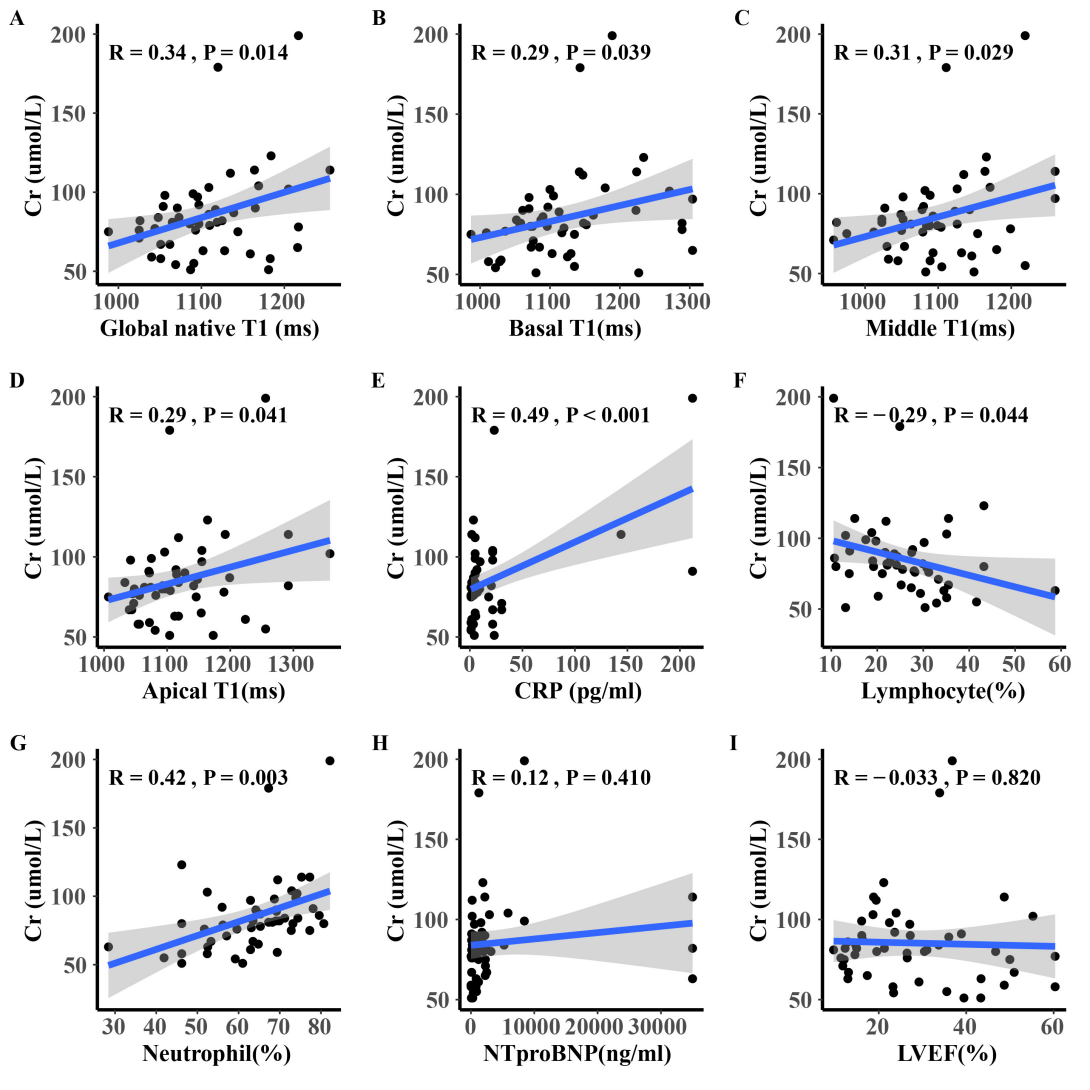
**Scatterplots (A to I) comparing serum creatinine and cardiac T1 (A,B,C,D), CRP, lymphocyte ratio, neutrophil ratio, NT-proBNP and LVEF**. Pearson correlation was adopted. CRP, c-reactive protein; NT-proBNP, N-terminal pro-B type natriuretic peptide; LVEF, left ventricle ejection fraction; Cr, serum creatinine.

Asymptomatic heart failure patients with elevated creatinine level and CRP level received cardiovascular magnetic resonance imaging and the results demonstrated a lesion in the cardiac (late gadolinium enhancement in the middle segment of inter-ventricular septum in short-axis view) (Fig. 2). The correlation between creatinine and the cardiac global native T1 was shown in Fig. 3. Serum creatinine level was significantly correlated with cardiac T1 (R = 0.34, *p* < 0.014), both in global and segmented analysis. A moderate correlation was observed in myocardial global T1 (R = 0.34, *p* = 0.014). Besides, there was a mild correlation between creatinine and inflammation marker (CRP R = 0.49, *p* < 0.001; lymphocyte R = –0.29, *p* < 0.044; Neutrophil R = 0.42, *p* = 0.003). Both LVEF and NT-proBNP were not significantly correlated with creatinine.

Table 2 summarizes the results of linear regression analysis for determinants of creatinine in all HF patients. Univariate analysis identified global native T1 (β = 0.16, 95% confidence interval (CI): 0.04–0.28, *p* = 0.014) and CRP (β = 0.30, 95% CI: 0.15–0.45, *p* < 0.001) as determinants of creatinine when age and diabetes were also screened. Multivariable linear regression analysis identified global native T1 (β = 0.12, 95% CI: 0.01–0.123, *p* < 0.040) as the determinant of creatinine while age and diabetes were adjusted.

**Table 2.** **Linear regression analysis of serum creatinine**.

	Univariate			Multivariable		
	β	95% CI	*p*	β	95% CI	*p*
Age	0.57	0.07~1.07	0.029	0.38	–0.07~0.83	0.100
Sex	–6.52	–25.02~11.98	0.493			
Diabetes	21.4	3.12~39.67	0.026	8.05	–9.27~25.37	0.354
Coronary artery disease	14.1	–0.89~29.10	0.071			
Atrial fibrillation	–12.27	–32.01~7.47	0.229			
Stroke	18.85	–13.14~50.83	0.254			
Body mass index	–0.82	–2.56~0.91	0.358			
CRP	0.3	0.15~0.45	<0.001	0.24	0.09~0.40	0.003
Global native T1	0.16	0.04~0.28	0.014	0.12	0.01~0.23	0.040
Extracellular volume	0.41	–0.37~1.19	0.306			
LV EF	–0.06	–0.62~0.49	0.82			
RV EF	0.36	–0.13~0.85	0.154			
LA volume (mL)	–0.05	–0.16~0.06	0.356			
LV GCS	1.22	–0.50~2.94	0.171			
LV GRS	–0.45	–1.38~0.49	0.355			
LV GLS	1.46	–0.45~3.37	0.140			
LGE	10.2	–6.71~27.10	0.243			

CRP, C-reactive protein; LV, left ventricle; EF, ejection fraction; RV, right ventricle; LA, left atrium; LGE, late gadolinium enhancement; GCS, global circumferential strain; GRS, global radial strain; GLS, global longitudinal strain.

**Fig. 4.**
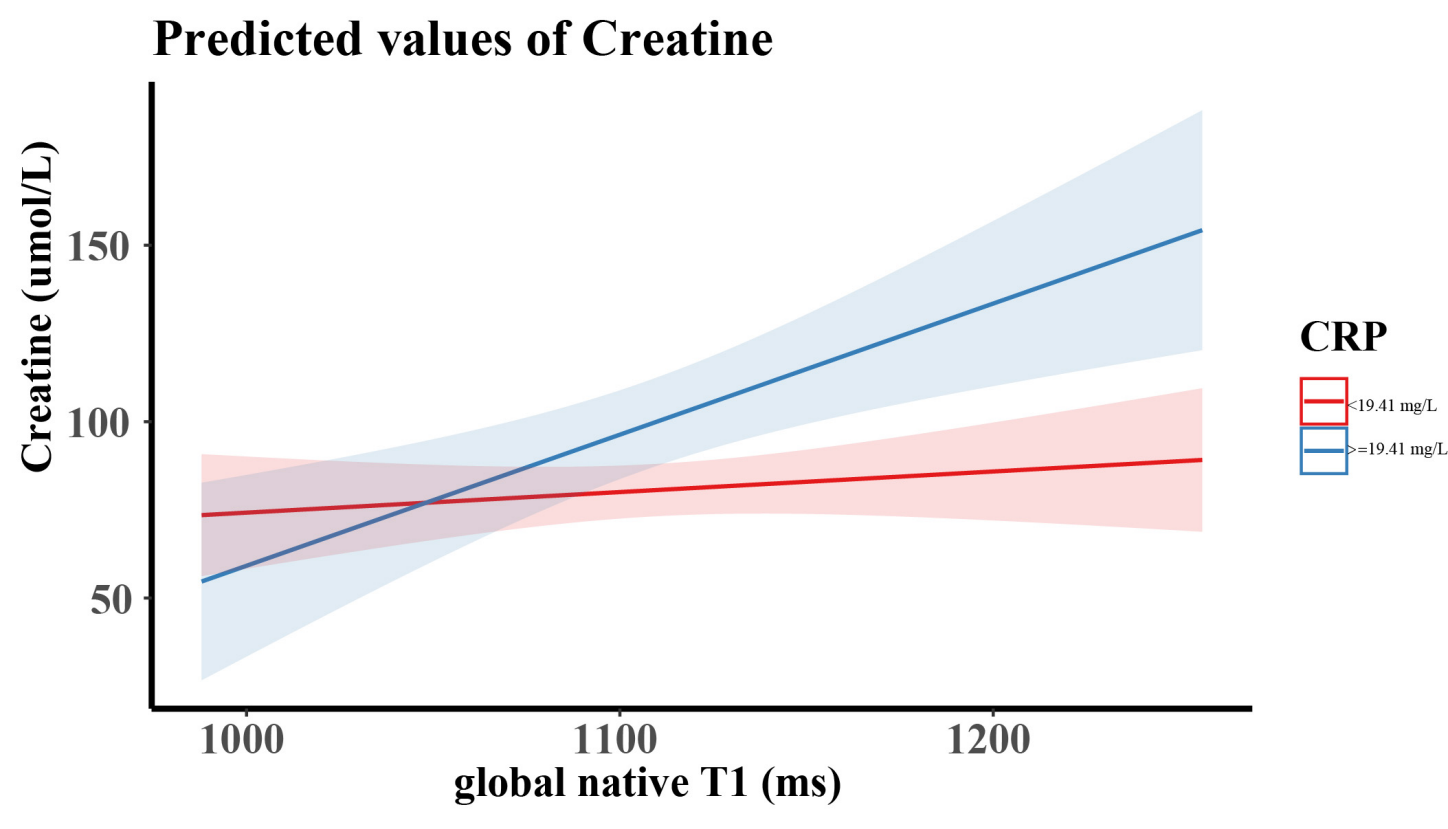
**Predicted probabilities of serum creatinine based on the interaction between CRP and cardiac native T1**. CRP was classified into two categories according to the mean value of CRP. CRP, C-reactive protein.

In order to analyze the association between CRP and native T1, an interaction analysis was performed (Fig. 4). We grouped the strata factors, which were classified into two categories (according to the mean of CRP): low (CRP <19.41 mg/L), and high levels (CRP ≥19.41 mg/L). Significant interactions between CRP and global native T1 in relation to creatinine levels (*p* for interaction = 0.005) were identified. The interaction tests for age and diabetes were not significant (*p* for interaction 0.352, 0.969 respectively).

### 3.3 Reproducibility

T1 mapping showed excellent intra-observer agreement: native T1: ICC = 0.998, 95% CI: 0.998–0.998; ECV: ICC = 0.992, 95% CI: 0.733–0.980.

## 4. Discussion

In this retrospective study, we demonstrate associations between creatinine levels and cardiac native T1. Native T1 was significantly associated with worsening kidney function. A serological marker of creatinine was associated with native T1 and CRP respectively. A significant interaction between CRP and native T1 was observed in different creatinine levels. According to these results, the interaction between myocardial injury and kidney dysfunction is contingent on the severity of the inflammatory response.

Our research provided clinical evidence that heart failure is associated with worsening kidney dysfunction. Native T1 was sensitive to myocardial fibrosis, edema, and iron overload. A previous cardiovascular magnetic resonance imaging study reported that native T1 (β = 0.125, *p* = 0.019) and T2 (β = 0.272, *p* = 0.001) were associated with eGFR [13]. A similar association was observed in another large sample study [11]. There are several potential explanations for the elevated cardiac T1 in kidney dysfunction patients including increased transmural pressure, small-vessel coronary obstruction, endothelial dysfunction, intracellular edema, and myocardial fibrosis [15, 16, 17]. Besides, hypotension during heart failure resulted in organ hypoperfusion, which might eventually contribute to kidney damage. It was reasonable to believe that elevated cardiac native T1 (represented cardiac damage) was associated with worsening kidney dysfunction.

This research extended the current understanding of cardiorenal syndrome. We provided evidence that myocardial damage (native T1 elevation) interacted with inflammation response in relation to kidney dysfunction. The association between myocardial damage and kidney dysfunction was less significant among individuals with low CRP levels compared to those with high levels. This phenomenon could be explained by cardiorenal syndrome, a bi-directional connection. A previous study demonstrated that inflammation contributed to the pathogenesis of cardiorenal syndrome [18]. Inflammatory biomarkers of CRP are known to predict worseoutcomes in cardiovascular and chronic diseases [19, 20, 21]. Various factors such as fluid retention, oxidative stress, obesity, smoking, and genetic factors contribute to this inflammation [4, 5]. Biomarkers of inflammation such as CRP pentraxin-3, IL-10, and IL-6 are associated with adeclining renal function [7, 22]. Besides, the inflammatory response plays a crucial role in vasculopathy and tissue remodeling in heart and kidney dysfunction [4, 23, 24]. Several potential biomarkers have been identified as practical tools for the assessment of cardiorenal syndrome, including native T1, a surrogate cardiac image biomarker. Native T1 is one of the parameters provided by cardiovascular T1 mapping. Besides, previous studies have shown that extracellular volume, another parameter of T1 mapping, is associated with a worse prognosis in heart failure patients [25, 26].

Although T1 mapping has been extensively studied, we discovered the usefulness of elevated native T1 as a biomarker for cardio-renal syndrome instead of ECV. A similar result was reported by a meta-analysis which showed that in the diagnosis of myocarditis, the area under curve (AUC) for T1 mapping was 0.95 (95% CI: 0.93 to 0.97), for ECV 0.81 (95% CI: 0.78 to 0.85), for LGE 0.87 (95% CI: 0.84 to 0.90) [27]. Accordingly, in diffuse amyloidosis cardiac damage, native T1 demonstrated a similar diagnostic value [28]. A possible explanation is that LGE is a quantifiable parameter that cannot reflect diffuse fibrosis, while ECV carries multiple measurement errors. Besides, a previous study found an independent association between native T2 and hs-cTnT in patients with severe CKD (eGFR <29 mL/min/1.73 $m^{2}$) [13]. According to the recommendation, T2 mapping serves as a sensitive tool in detecting edema; T1 mapping is useful in detecting infiltration, fibrosis, and acute injury cardiac disease [29]. Renal function affects the rate of gadolinium deposition; hence, the use of a gadolinium agent has been limited in kidney dysfunction. Therefore, incorporating quantitative native T1 assessment into routine CMR evaluations provides incremental risk stratification in heart failure through the detection of cardiorenal syndrome.

### Limitation

First, this study was a small sample, retrospective study. A further prospective, large cohort study would prove the diagnostic and prognostic value of inflammation in the cardiorenal syndrome. Second, it would be desirable to include measurements such as T2 mapping, and T2* mapping and proteinuria at the original design to fully characterize tissue of cardiac and kidney, and help understand the connection of cardiorenal syndrome; however, due to the retrospective design, there is limited data when parameter mapping was not commonly adopted in the clinical practice. Thirdly, tissue biopsy would serve as the gold standard for myocardial and renal pathological changes, and provide solid evidence for the theory of inflammation-driven cardiorenal syndrome. We aim to discuss this issue in future studies.

## 5. Conclusions

This study demonstrates myocardial inflammation and fibrosis assessed by CMR correlate with renal dysfunction in heart failure patients. T1 mapping identifies myocardial injury associated with elevated inflammatory markers and renal impairment. Cardiac inflammation likely mediates the link between cardiomyopathy and kidney disease.

## Data Availability

The datasets used and/or analyzed during the current study are available from the corresponding author on reasonable request.
